# Primary pulmonary plasmacytoma: A case report

**DOI:** 10.1097/MD.0000000000040941

**Published:** 2024-12-20

**Authors:** Cong Jin, Lina Yan, Jing Zhang, Lihua Cao, Dan Zhang

**Affiliations:** a Department of Respiratory and Critical Care Medicine, The Second Hospital of Dalian Medical University, Dalian, Liaoning Province, China.

**Keywords:** extramedullary, lung, plasmacytoma, pulmonary

## Abstract

**Rationale:**

Extramedullary plasmacytoma is an extremely rare malignant clonal plasmacytoma, which can occur in any tissue or organ of the body other than bone marrow hematopoietic tissue. Lesions mostly occur in the head and neck or upper respiratory tract, and rarely in the lower respiratory tract. Primary plasmacytoma of the lung, also known as primary pulmonary plasmacytoma (PPP), is rarer and is mainly diagnosed on the basis of the histopathology of biopsy tissue. In this report, we present a case of PPP.

**Patient concerns:**

A 65-year-old woman with repeated episodes of cough and sputum for many years was hospitalized for an acute episode of chest pain and shortness of breath. Chest computed tomography revealed bilateral inflammation of multiple lobes, accompanied by slight bronchial dilatation.

**Diagnoses:**

The results of pathology of the electronic bronchoscopy sample were different from those of common infections but were consistent with plasmacytoma.

**Interventions and outcomes:**

The patient was discharged after hormone anti-inflammatory therapy. As patients lose contact, further follow-up of the patient was not possible.

**Lessons:**

PPP is a rare lung tumor, and diagnosis of this lesion on the basis of bronchoscopic biopsy is even rarer. With this case report, we seek to enhance the awareness and understanding of PPP. We recommend that in cases wherein chest imaging findings suggest multiple lung lesions, the possibility of pulmonary plasmacytoma should also be considered, and biopsy should be actively performed to confirm the diagnosis and guide treatment. This report provides valuable insights and guidance for clinicians in the management and diagnosis of patients with PPP.

## 1. Introduction

Extramedullary plasmacytoma (EMP) is a monoclonal plasmacytoma originating outside the bone marrow and hematopoietic tissue. It can occur in any extramedullary tissue or organ and is relatively rare in clinical practice. Most lesions occur in the head and neck or upper respiratory tract, and lesions in the lower respiratory tract are rare.^[1]^ Primary pulmonary plasmacytoma (PPP) is an extremely rare type of EMP. The clinical symptoms and imaging findings of PPP are nonspecific, whereby it can be easily misdiagnosed. The diagnosis mainly relies on histopathology of biopsy tissue.^[2]^ Here, we report a rare case of PPP and review the relevant literature.

## 2. Case presentation

**Figure 1.**
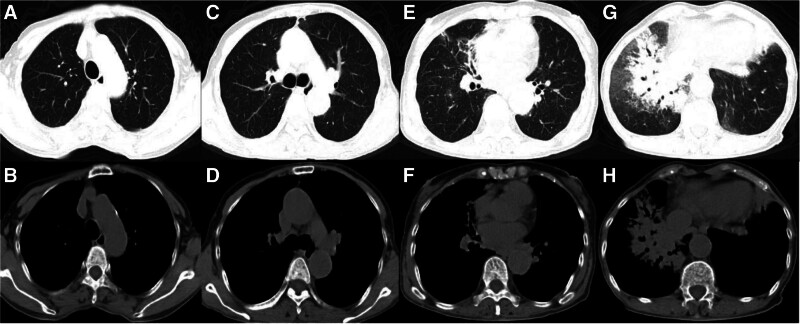
2021-05-25 chest CT: (A–H) show slight dilation of the bronchus in the middle lobe of the right lung, the upper lobe of the left lung, and the lower lobe of both lungs, and the shadow of the surrounding spots, significant in the middle and lower lobe of the right lung, partial lung tissue consolidation, visible air bronchus, fuzzy nodules in both lungs, calcified nodules in the right subpleura, and calcification in the aorta and coronary walls. CT = computed tomography.

A 65-year-old woman who had a history of cough and sputum for 40 years was admitted to the hospital in May 2021 for chest pain since 10 days. The patient had a history of repeated hemoptysis, cough, and sputum and was previously diagnosed with bronchiectasis at another hospital. The patient had a history of enlarged lymph nodes 25 years ago, which was diagnosed as chronic lymphangitis. Six years before the presentation, she was diagnosed with osteoarthritis. Chest computed tomography (CT) performed following a previous outpatient visit revealed multilobar inflammatory changes in both lungs, accompanied by slight bronchial dilation, multiple nodules in both lungs, and tracheal diverticulum (Fig. 1). On admission, vital signs were as follows: blood pressure of 120/73 mm Hg, pulse rate of 104 times/minute, respiratory rate of 22 times/minute, and body temperature of 36.5°C. Physical examination did not show enlargement of any superficial lymph nodes, while auscultation revealed coarse breathing sounds in both lungs, with no signs of dry or wet rales.

**Figure 2.**
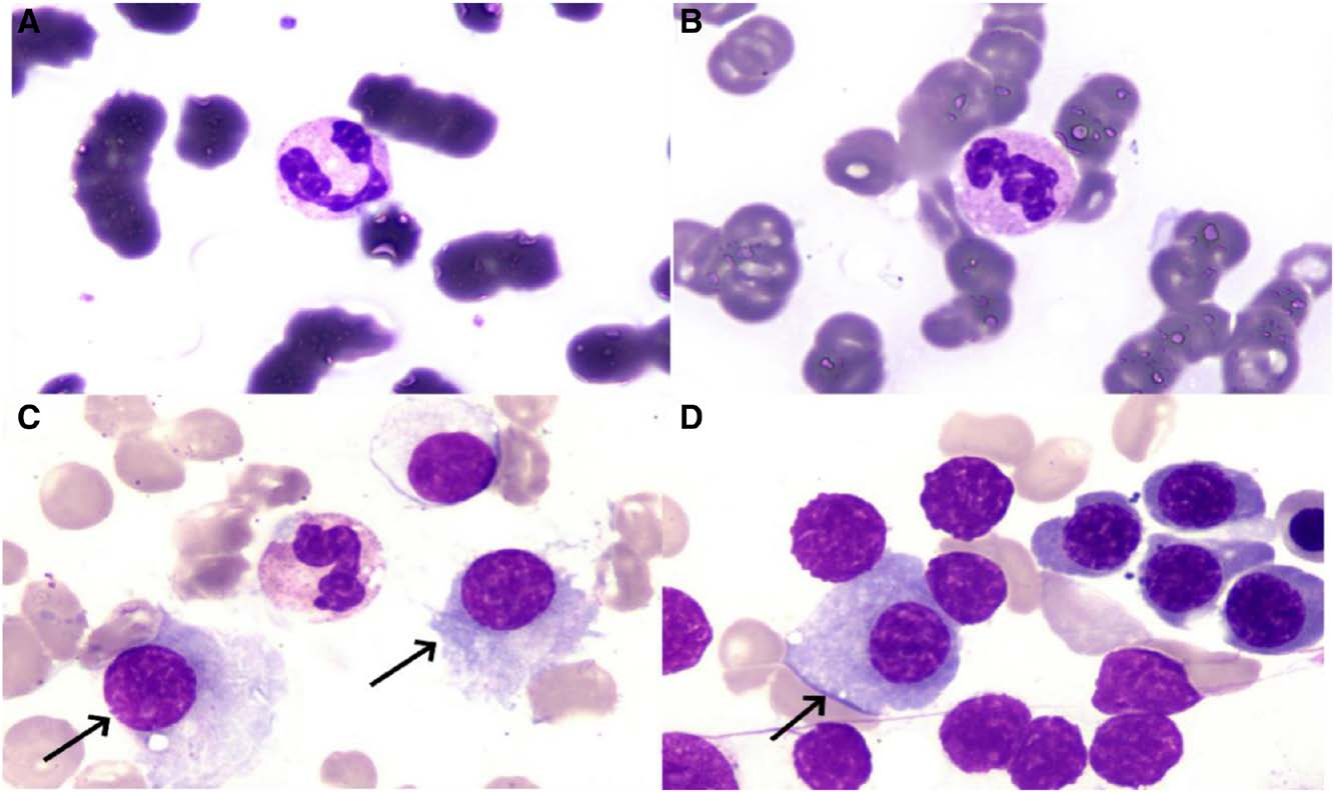
(A, B) are peripheral blood smears showing leukopenia, rouleaux arrangement of mature red blood cells, low platelet count, scattered, small piles. (C, D) are bone marrow smears showing panhemocytopenia. There are occasional naive plasma cells in the bone marrow. The black arrows indicate plasma cells.

**Figure 3.**
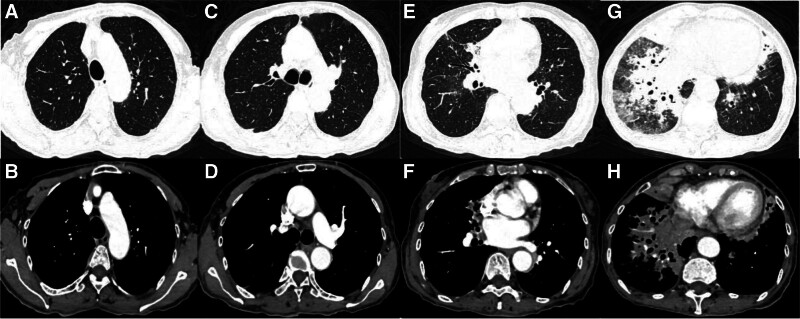
2021-05-30 enhanced scan with thin-section CT: (A, C, E) and (G) were pulmonary windows. The bronchi of the middle lobe of the right lung, the lingual segment of the upper lobe of the left lung, and the lower lobe of both lungs were slightly dilated; the tube wall was thickened; and the surrounding spots were seen, significant in the middle and lower lobes of the right lung; some lung tissue was solid; air bronchi were visible; and vague nodules were seen in both lungs. (B, D, F) and (H) were enhanced mediastinal windows. After enhancement, slight enhancement of solid tissue was observed; calcified nodules were observed under the right pleura; and calcified aorta and coronary wall were observed. CT = computed tomography.

**Figure 4.**
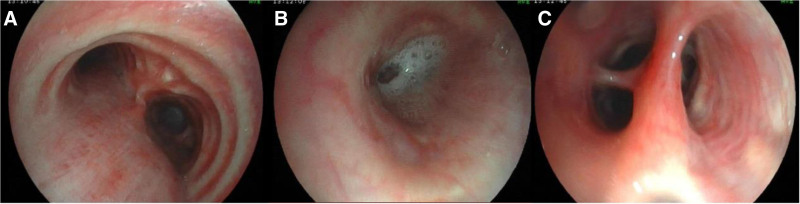
Tracheoscopy: (A) is the bulge; (B) is the opening of the right middle bronchus with a small amount of purulent secretion. (C) is the basal branch of the right inferior lobe.

**Figure 5.**
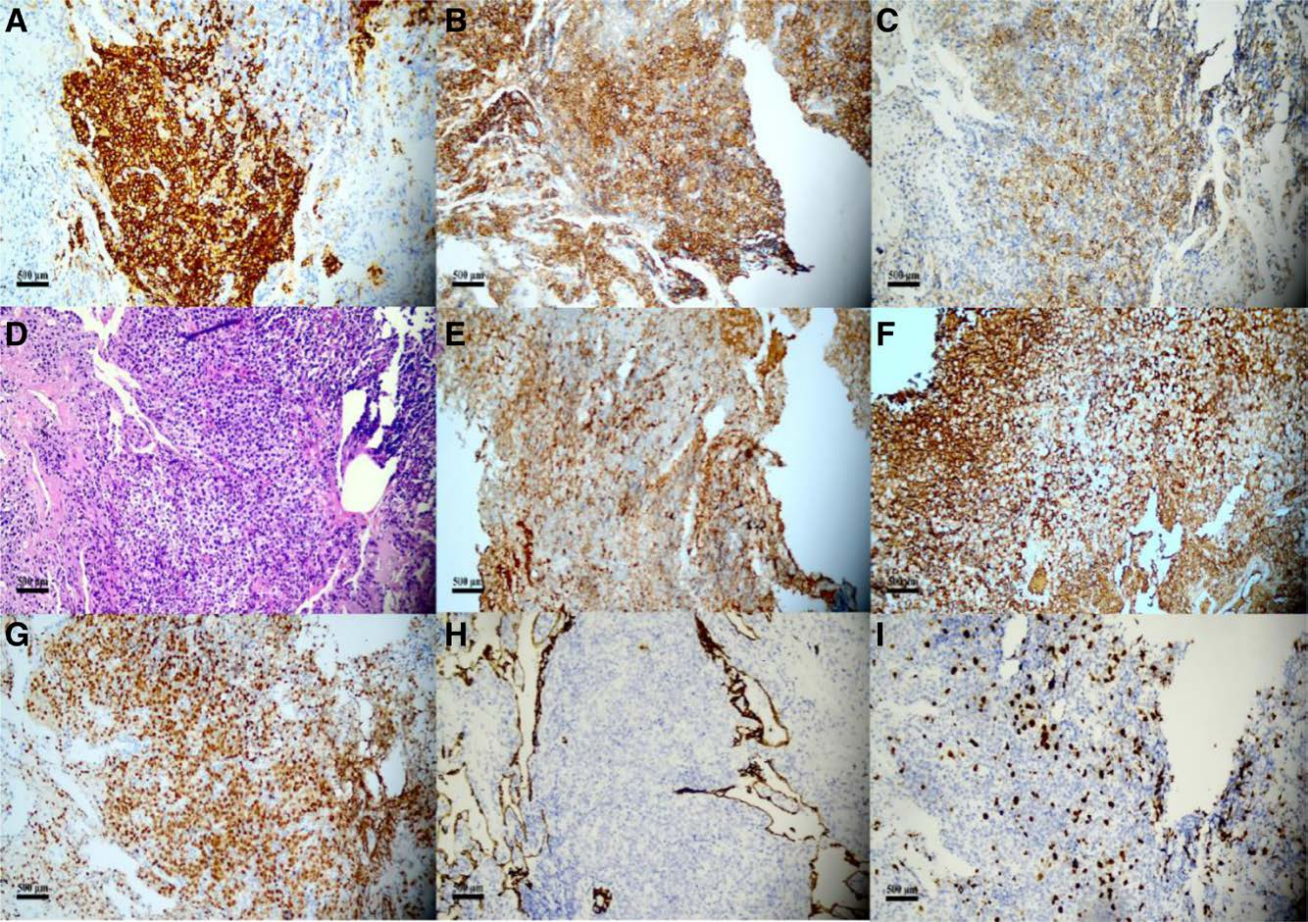
(A–I) Cells of the same size and shape were dispersed and the nuclei were displaced, which was consistent with plasma cell tumor when combined with the findings of immunohistochemical results: (A) CD20; (B) CD38; (C) CD138; (D) HE; (E) Kappa; (F) Lambda; (G) MUM1; (H) AE1/AE3; (I) Ki-67 (magnification ×200).

Improvement was seen in relevant tests after hospitalization (Table 1). Bone marrow biopsy showed pancytopenia, with occasional naive plasma cells; juvenile plasma cells accounted for 0.5% (normal range, 0.00–0.23%) and mature plasma cells accounted for 1% (normal range, 0.01–1.50%; Fig. 2). Immunotyping was negative for myeloma. Contrast-enhanced CT of the chest showed scattered inflammation, partial consolidation, bronchiectasis, and infection in both lungs, with multiple nodules in both lungs and tracheal diverticulum (Fig. 3). Electronic bronchoscopy showed a small amount of purulent discharge at the bronchial opening of the right inferior lobe (Fig. 4). Pathologic examination of the biopsy sample showed that cells with uniform size and shape were dispersed locally and the nuclei were displaced, which are findings consistent with those of plasmacytoma. The results of immunohistochemistry of the tumor cells were as follows: CD20 (+), CD79a (+), CD38 (+), CD138 (+), MUM1 (+), Kappa (+), Lambda (+), CD56 (−), CD3 (−), CD5 (−), AE1/AE3 (epithelial cells +), and Ki-67 (20%+; Fig. 5). On the basis of the above results, the diagnosis of plasmacytoma was made, with need for hematology consultation.

**Table 1:** Summary of blood test results.

Inspection data	Value	Unit	Normal reference range
White blood cell	2.5	10^9^/L	3.5–9.5
Red blood cell	2.56	10^12^/L	3.8–5.10
Hemoglobin	78	g/L	115–150
Blood platelet	98	10^9^/L	125–350
ESR	104	mm/h	0–20
APTT	57.50	s	26–43
APTT mixing test	57.40	s	26–43
Globulin (liver biochemistry)	83.34	g/L	20–40
Immunoglobulin IgA	41.40	g/L	0.7–4.0
Immunoglobulin IgG	29.10	g/L	7–16
Immunoglobulin IgM	0.32	g/L	0.4–2.3
Blood free light-chain KAP	151.000	mg/L	6.7–22.4
Blood free light-chain LAM	32.300	mg/L	8.3–27.0
Blood light-chain KAPPA (KAP)	14.600	g/L	1.7–3.7
Blood light-chain LAMBDA (LAM)	5.180	g/L	0.9–2.1
KAP/LAM	2.819		1.35–2.65
Urine light-chain KAPPA (KAP_-_U)	384.000	mg/L	0–7.1
Urine light-chain LAMBDA (LAM_-_U)	20.100	mg/L	0–4.0
KAP_-_U/LAM_-_U	19.104		0.75–4.50
Immunofixed typing IgA	Positive		Negative
Immunofixed typing κ chain	Positive		Negative

APTT = activated partial thromboplastin time, ESR = erythrocyte sedimentation rate.

During the hospitalization, the patient developed soy-colored urine, but no stones or tumors were detected on ultrasound examination of the urinary system. Urine routine showed urocholinogen (−), urinary protein (+), urinary occult blood (+++), urinary red blood cell count of 3815.70/hpf (normal range: 0.00–2.27/hpf). The following assessments were made to check for disseminated intravascular coagulation: antithrombin III was 59.00% (normal range: 75–125%), prothrombin time was 16.60 seconds (normal range, 11–14.5 seconds), activated partial thrombin time was 55.40 seconds (normal range, 26–43 seconds), and fibrinogen content was 1.82 g/L (normal range: 2–4 g/L). Kidney function test, sucrose test, Hams test, and Coombs test showed no obvious abnormality; hemoglobin did not decrease; and the results of 2 bilirubin tests were normal. Hematology consultation was considered to rule out hemolysis caused by the plasmacytoma, and a transfer to the hematology department for further diagnosis and treatment was recommended. However, the patient and her family refused the transfer. Finally, hematology consultation was sought, and the patient was allowed to be discharged from the hospital after hormone anti-inflammatory therapy. Following discharge, the patient did not visit our hospital regularly for follow-up and treatment adjustment, and we were unable to contact the patient or her family. Thus, further follow-up of the patient was not possible.

## 3. Discussion and conclusions

Plasmacytoma is a monoclonal hyperplastic tumor originating from B lymphocytes, and it mainly occurs in soft tissues or organs. Clinically, 4 main types are recognized: multiple myeloma, solitary plasmacytoma of bone, EMP, and plasma cell leukemia. EMP is a rare malignant tumor that can occur anywhere in the body, but very rarely occurs in the lower respiratory tract. There are 2 main subtypes of EMP: primary EMP, which occurs without bone marrow involvement and secondary EMP, which occurs with previous bone marrow involvement.^[3]^ PPP is a type of EMP, which is an extremely rare malignant tumor in the lung.

The pathogenetic processes underlying EMP are unclear, although some investigators believe that EMP originates from malignant primordial plasma cells. PPP can occur at any age, and most patients are between 40 and 70 years old. The proportion of male and female cases is almost similar (13:11).^[4]^ The clinical manifestations of EMP are varied, but lack specificity. The manifestations are mainly related to the regions of invasion. Involvement of the nasopharynx may lead to symptoms such as epistaxis, abnormal smell, and headache, whereas involvement of the lung may cause cough, sputum, shortness of breath, and hemoptysis. Pleural involvement may cause chest pain, whereas peritoneal involvement may lead to abdominal pain. On the other hand, some patients may have no obvious clinical symptoms.^[5]^

Imaging findings are generally atypical, comprising a round or oval smooth lesion with clear boundary and uniform texture of the lump. The lesion is mostly located around the hilus of the lung, with no obvious necrosis and calcification.^[6,7]^ Other manifestations of the disease include multiple nodules and mass shadows in the lungs or obstructive pneumonia changes and diffuse infiltrative lesions, but these manifestations are relatively rare.^[8]^

Contrast-enhanced chest CT in PPP usually shows moderate to significant enhancement and abnormally increased vascular shadows in and around the diseased lesion.^[9,10]^ These findings need to be differentiated from those of lung cancer. Central lung cancer is associated with hilar mass and bronchial changes, thickening of the bronchial wall, and possible obstructive emphysema, obstructive pneumonia, obstructive atelectasis, mucous impaction, pulmonary vascular changes, pleural effusion, and hilar mediastinal lymph node enlargement. On the other hand, peripheral lung cancer is associated with the presence of vacuolar signs, air bronchogram signs, calcification, necrosis, liquefaction, or cavity formation inside the tumor.^[11]^ As a special form of peripheral lung cancer, pneumonic lung cancer is mainly associated with ground-glass opacity of segments of the lung lobes, and it is one of the main differential diagnoses in this patient.^[12]^

The diagnosis of PPP is mainly based on the pathologic findings, which show diffuse infiltration of plasma cells^[13]^ and exclude the primary bone marrow lesion. Monoclonal plasma cells are detected on pathological examination and immunohistochemical markers, including CD138 (+), CD79a (+), and CD20 (−), are detected.^[14]^

At present, there are no unified diagnostic criteria for PPP, but some reports suggest that the diagnosis of this disease should be considered if the following conditions are met: isolated plasma cell tumor in the lung is confirmed by biopsy (sometimes the tumor is considered as an isolated tumor even when it involves adjacent bone structures or surrounding lymph nodes); bone marrow aspirate shows normal bone marrow architecture; proportion of marrow plasma cells is < 5%; and Other conditions, namely, lung cancer, pulmonary tuberculosis, pneumonia, plasma cell granuloma, lymphoplasmacytic lymphoma, Waldenstrom macroglobulinemia, and other pulmonary diseases are ruled out.^[15]^

In this case, the patient had a history of cough, sputum, shortness of breath, and chest pain, with initial symptoms being attributed to bronchiectasis. However, the patient had no fever, c-reactive protein and procalcitonin were normal, and chest CT showed inflammation in multiple lobes of both lungs, with the lesions in the right lung being more severe and accompanied by slight bronchodilation. The bronchodilatory changes were not very typical or serious, and the bilateral pulmonary lesions, especially the lesions in the lower lobe of the right lung, could not be explained by pulmonary infection. Further, electronic bronchoscopy showed that the pathology was consistent with plasma cell tumor. The diagnosis of PPP is clearly established through lung biopsy or surgical pathology; it is only in very few cases that the chest CT does not show any clear space-occupying lesion and the lesion is found through bronchoscopy, as observed in the present case. Only Montero et al^[16]^ have reported similar findings of mucosal infiltration detected under bronchoscopy with presence of polypoid lesions, followed by a diagnosis of PPP after biopsy. Establishing the definitive diagnosis through electronic bronchoscopy is safe, technically simple, and easy for patients and their families to accept, and it has less adverse reactions. During hospitalization, the patient developed abnormal urine color and subsequent urine routine indicated hematuria. The hematuria was considered to be caused by the primary disease of plasmacytoma, but the patient refused further treatment in the hematology department and was allowed to be discharged after receiving hormone therapy. Unfortunately, the patient did not receive regular treatment and follow-up at our hospital, and the patient and his family could not be contacted. Therefore, we are unable to evaluate the long-term outcome or prognosis of the condition or treatment efficacy.

If there are indications for surgery after PPP is diagnosed, surgery should be performed as soon as possible to completely remove the lesion, because it may be possible to achieve clinical cure. However, some patients may still require radiotherapy and chemotherapy,^[17]^ but the efficacy of such treatment has not yet been established. Radiotherapy doses administered in most cases are 4000~5000 cGy, and the local control rate is 80~90%.^[18]^ In addition, some patients may also require adjuvant chemotherapy depending on their general condition. Generally, the chemotherapy regimen administered is similar to that used in multiple myeloma. Other alternative therapeutic agents that may be considered in the treatment of PPP are traditional drugs (such as magfaran and dexamethasone), protease inhibitors (such as bortezomib and ixazomib), and some immunomodulators (such as thalidomide and pomadomide). The therapeutic outcome mainly depends on factors such as the age of the patient and presence/absence of complications. However, due to the rarity of the disease, data on therapeutic effects are still lacking.^[18]^ Compared with other lung malignancies, PPP has a relatively good prognosis, and most patients can achieve long-term survival. The patient’s records did show a history of lymph node enlargement several decades ago, although it is not clear whether this was related to this disease. If it is indeed related to the disease, the disease may have lasted for a long time; however, the patient had no enlargement of lymph nodes at presentation, so lymph node biopsy was not performed. In patients with EMP, the presence of malignant pleural effusion suggests a poor prognosis. According to the available literature, 61.1–64.7% of all treated EMP patients did not have a relapse or progress to multiple myeloma, while 21.2–22.0% had a relapse, and 14.1–16.1% developed multiple myeloma.^[19]^ Due to the possibility of recurrence and development of multiple myeloma in PPP and EMP, long-term close follow-up is necessary.

In summary, PPP is a rare lung tumor characterized by a lack of typical clinical manifestations in most patients and specific imaging findings; therefore, it is relatively difficult to diagnose, and often, diagnosis and treatment are delayed. From our experience in this case, we recommend that when chest imaging is suggestive of multiple lung lesions, the possibility of plasmacytoma should be considered after excluding common diseases. In such cases, a comprehensive evaluation and early biopsy are required to make a clear diagnosis and guide further treatment.

## Author contributions

**Conceptualization:** Cong Jin, Lina Yan.

**Data curation:** Cong Jin, Jing Zhang.

**Formal analysis:** Cong Jin, Lihua Cao, Dan Zhang.

**Investigation:** Cong Jin, Dan Zhang.

**Methodology:** Cong Jin, Jing Zhang.

**Project administration:** Cong Jin.

**Resources:** Cong Jin, Lihua Cao.

**Software:** Cong Jin, Dan Zhang.

**Supervision:** Cong Jin, Lihua Cao.

**Validation:** Cong Jin, Lina Yan.

**Visualization:** Cong Jin, Dan Zhang.

**Writing – original draft:** Cong Jin, Lina Yan, Jing Zhang.

**Writing – review & editing:** Cong Jin, Lina Yan, Jing Zhang, Lihua Cao, Dan Zhang.

## References

[1] Galieni P Cavo M Pulsoni A. Clinical outcome of extramedullary plasmacytoma. Haematologica. 2000;85:47–51. 10629591

[2] Joseph G Pandit M Korfhage L. Primary pulmonary plasmacytoma. Cancer. 1993;71:721–4. 8431851 10.1002/1097-0142(19930201)71:3<721::aid-cncr2820710311>3.0.co;2-u

[3] Alexiou C Kau RJ Dietzfelbinger H. Extramedullary plasmacytoma: tumor occurrence and therapeutic concepts. Cancer. 1999;85:2305–14. 10357398

[4] Zhou NK tian XD Sun YE. A case of primary pulmonary plasmacytoma. J Chin PLA Postgraduate Med School. 2001;22(:314–314.

[5] Zhang T Cai JL Yu J. A case of primary pulmonary plasmacytoma and literature review. JiangxiMedicine. 2021;56:611–3.

[6] Wang TT Dong JN Lin TT. Analysis of the imaging features in patients with extramedullary plasmacytoma in head and neck region and thorax. Chinese Journal of ctandmri. 2018;16:30–2.

[7] Nie S Peng DC Li HJ. A case of primary pulmonary plasmacytoma. J Clin Radiol. 2017;36:300.

[8] Kim SH Kim TH Sohnj W. Primary pulmonary plasmacytoma presenting as multiple lung nodules. Korean J Intern Med. 2012;27:111–3. 22403510 10.3904/kjim.2012.27.1.111 PMC3295978

[9] Egashira K Hirakata K Nakata H. CT and MRI manifestations of primary pulmonary plasmacytoma. Clin Imaging. 1995;19:17–9. 7895190 10.1016/0899-7071(94)00033-9

[10] Kaneko Y Satoh H Haraguchi N Imagawa S Sekizawa K. Radiologic findings in primary pulmonary plasmacytoma. J Thorac Imaging. 2005;20:53–4. 15729124 10.1097/01.rti.0000139389.88019.63

[11] Zhangliu G Hongzhen Y. Imaging diagnosis and new progress of lung cancer. Chin J Pract Med. 2008;35:94–5.

[12] Yuqing S Xian L Wangyan Z Xurui B. MSCT imaging features and pathological comparison of pneumonic lung cancer. Chin J Pract Med. 2011;38:49–51.

[13] Wang S Chen H Jiang YJ. Clinicopathologic characteristics of solitary extramedullary plasmacytoma: analysis of 15 cases. J Leukemia lymphoma. 2016;25:747–51.

[14] Zhou GY Gao BQ Liu YF. Extramedullary plasmacytoma of the head and neck: a clinicopathologic, immunohistochemistry study. J Clin Otorhinolaryngol (China). 2000;14:168–70. 12541493

[15] Li KL Kang J Liao BH. One case of primary pulmonary plasmacytoma. Modern Oncology. 2011;19:2006–8.

[16] Montero C Souto A Vidal I. Three cases of primary pulmonary plasmacytoma. Archivos De Bronconeumología. 2009;45:564–6. 19523733 10.1016/j.arbres.2009.04.009

[17] Sunkara T Sharma SR Ofosu A Gaduputi V Reddy M Shahzad G. A case of concurrent gastric and pancreatic plasmacytomas in a patient with multiple myeloma: an extremely rare entity. J Invest Med High Impact Case Rep. 2018;6. 10.1177/2324709618777003 PMC5971378 29854857

[18] Caers J Paiva B Zamagni E. Diagnosis, treatment, and response assessment in solitary plasmacytoma: updated recommendations from a European Expert Panel. J Hematol Oncol. 2018;11:10. 29338789 10.1186/s13045-017-0549-1 PMC5771205

[19] Park JI Lee YY Lee SS Ahn JH. A rare case of primary solitary endobronchial plasmacytoma. Thoracic Cancer. 2021;12:958–61. 33501775 10.1111/1759-7714.13853 PMC7952851

